# 2-[2-(Cyclo­hexyl­carbon­yl)phen­yl]-1-phenyl­ethanone

**DOI:** 10.1107/S1600536809041270

**Published:** 2009-10-17

**Authors:** F. Nawaz Khan, P. Manivel, K. Prabakaran, Venkatesha R. Hathwar, Seik Weng Ng

**Affiliations:** a Chemistry Division, School of Science and Humanities, VIT University, Vellore 632 014, Tamil Nadu, India; b Solid State and Structural Chemistry Unit, Indian Institute of Science, Bangalore 560 012, Karnataka, India; c Department of Chemistry, University of Malaya, 50603 Kuala Lumpur, Malaysia

## Abstract

The title diketone, C_21_H_22_O_2_, features a phenyl­ene ring having benzoyl­methyl and cyclo­hexa­noyl substituents *ortho* to each other. The cyclo­hexyl ring adopts a chair conformation with the ketonic group occupying an equatorial position; the four-atom –C(O)–C ketonic unit is twisted out of the plane of the phenyl­ene ring by 34.9 (1)°.

## Related literature

For the synthesis of this and other 1,2-phenyl­ethano­nes from isocoumarins, see: Manivel *et al.* (2008 ▶).
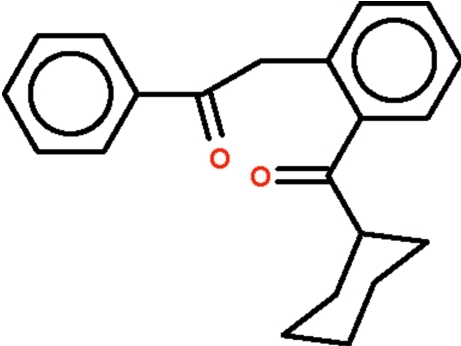


## Experimental

### 

#### Crystal data

C_21_H_22_O_2_
*M* *_r_* = 306.39
Monoclinic, 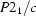

*a* = 10.4012 (6) Å
*b* = 10.1132 (6) Å
*c* = 16.0981 (9) Å
β = 90.038 (1)°
*V* = 1693.35 (17) Å^3^
*Z* = 4
Mo *K*α radiation
μ = 0.08 mm^−1^
*T* = 290 K
0.25 × 0.22 × 0.18 mm

#### Data collection

Bruker SMART area-detector diffractometer
Absorption correction: none
11930 measured reflections
2984 independent reflections
2797 reflections with *I* > 2σ(*I*)
*R*_int_ = 0.025

#### Refinement

*R*[*F* ^2^ > 2σ(*F* ^2^)] = 0.074
*wR*(*F* ^2^) = 0.183
*S* = 1.32
2984 reflections
208 parameters
H-atom parameters constrained
Δρ_max_ = 0.18 e Å^−3^
Δρ_min_ = −0.18 e Å^−3^

### 

Data collection: *SMART* (Bruker, 2004 ▶); cell refinement: *SAINT* (Bruker, 2004 ▶); data reduction: *SAINT*; program(s) used to solve structure: *SHELXS97* (Sheldrick, 2008 ▶); program(s) used to refine structure: *SHELXL97* (Sheldrick, 2008 ▶); molecular graphics: *X-SEED* (Barbour, 2001 ▶); software used to prepare material for publication: *publCIF* (Westrip, 2009 ▶).

## Supplementary Material

Crystal structure: contains datablocks global, I. DOI: 10.1107/S1600536809041270/tk2553sup1.cif

Structure factors: contains datablocks I. DOI: 10.1107/S1600536809041270/tk2553Isup2.hkl

Additional supplementary materials:  crystallographic information; 3D view; checkCIF report

## Figures and Tables

**Fig. 1.**
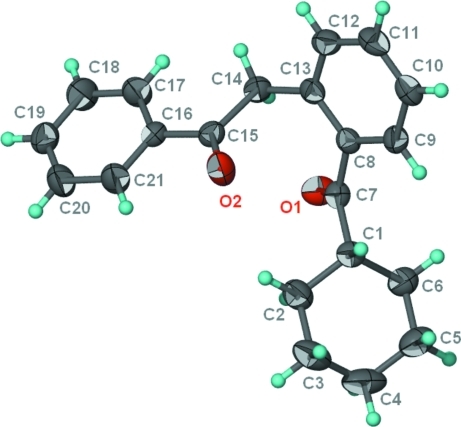
Thermal ellipsoid plot (Barbour, 2001) of C21H22O2 at the 50% probability level; hydrogen atoms are drawn as spheres of arbitrary radius.

## supplementary crystallographic information

### Experimental

The compound was synthesized as described by Manivel *et al.* (2008). Single crystals were grown from its solution in ether.

### Refinement

Carbon-bound H-atoms were placed in calculated positions (C–H 0.93–0.98 Å) and were included in the refinement in the riding model approximation with *U*_iso_(H) set to 1.2*U*_eq_(C).

### Crystal data

**Table:**

C_21_H_22_O_2_	*F*(000) = 656
*M**_r_* = 306.39	*D*_x_ = 1.202 Mg m^−^^3^
Monoclinic, *P*2_1_/*c*	Mo *K*α radiation, λ = 0.71073 Å
Hall symbol: -P 2ybc	Cell parameters from 878 reflections
*a* = 10.4012 (6) Å	θ = 2.4–25.3°
*b* = 10.1132 (6) Å	µ = 0.08 mm^−^^1^
*c* = 16.0981 (9) Å	*T* = 290 K
β = 90.038 (1)°	Block, colorless
*V* = 1693.35 (17) Å^3^	0.25 × 0.22 × 0.18 mm
*Z* = 4	

### Data collection

**Table:**

Bruker SMART area-detector diffractometer	2797 reflections with *I* > 2σ(*I*)
Radiation source: fine-focus sealed tube	*R*_int_ = 0.025
graphite	θ_max_ = 25.0°, θ_min_ = 2.0°
φ and ω scans	*h* = −12→12
11930 measured reflections	*k* = −12→12
2984 independent reflections	*l* = −18→19

### Refinement

**Table:**

Refinement on *F*^2^	Primary atom site location: structure-invariant direct methods
Least-squares matrix: full	Secondary atom site location: difference Fourier map
*R*[*F*^2^ > 2σ(*F*^2^)] = 0.074	Hydrogen site location: inferred from neighbouring sites
*wR*(*F*^2^) = 0.183	H-atom parameters constrained
*S* = 1.32	*w* = 1/[σ^2^(*F*_o_^2^) + (0.068*P*)^2^ + 0.5871*P*] where *P* = (*F*_o_^2^ + 2*F*_c_^2^)/3
2984 reflections	(Δ/σ)_max_ = 0.001
208 parameters	Δρ_max_ = 0.18 e Å^−^^3^
0 restraints	Δρ_min_ = −0.18 e Å^−^^3^

### Fractional atomic coordinates and isotropic or equivalent isotropic displacement parameters (Å^2^)

**Table:**

	*x*	*y*	*z*	*U*_iso_*/*U*_eq_	
O1	0.63291 (18)	0.81169 (19)	0.64919 (12)	0.0603 (6)	
O2	0.83697 (18)	0.5709 (2)	0.67129 (13)	0.0649 (6)	
C1	0.8136 (2)	0.9209 (2)	0.70629 (15)	0.0444 (6)	
H1	0.8661	0.9089	0.7563	0.053*	
C2	0.8988 (3)	0.8982 (3)	0.63032 (19)	0.0617 (8)	
H2A	0.9350	0.8099	0.6326	0.074*	
H2B	0.8472	0.9047	0.5803	0.074*	
C3	1.0069 (3)	0.9992 (4)	0.6269 (2)	0.0772 (10)	
H3A	1.0633	0.9868	0.6743	0.093*	
H3B	1.0570	0.9855	0.5769	0.093*	
C4	0.9553 (4)	1.1381 (4)	0.6276 (2)	0.0855 (11)	
H4A	0.9063	1.1536	0.5772	0.103*	
H4B	1.0264	1.2001	0.6285	0.103*	
C5	0.8704 (3)	1.1617 (3)	0.7022 (2)	0.0737 (9)	
H5A	0.8346	1.2501	0.6993	0.088*	
H5B	0.9216	1.1555	0.7524	0.088*	
C6	0.7621 (3)	1.0616 (3)	0.7059 (2)	0.0593 (7)	
H6A	0.7062	1.0736	0.6582	0.071*	
H6B	0.7116	1.0764	0.7556	0.071*	
C7	0.7044 (2)	0.8228 (2)	0.70871 (15)	0.0426 (6)	
C8	0.6826 (2)	0.7417 (2)	0.78542 (14)	0.0405 (6)	
C9	0.7074 (2)	0.7974 (3)	0.86283 (15)	0.0468 (6)	
H9	0.7392	0.8832	0.8655	0.056*	
C10	0.6862 (3)	0.7290 (3)	0.93556 (16)	0.0543 (7)	
H10	0.7033	0.7683	0.9866	0.065*	
C11	0.6397 (3)	0.6023 (3)	0.93171 (17)	0.0574 (7)	
H11	0.6245	0.5551	0.9803	0.069*	
C12	0.6154 (2)	0.5451 (3)	0.85552 (18)	0.0520 (7)	
H12	0.5841	0.4590	0.8538	0.062*	
C13	0.6361 (2)	0.6117 (2)	0.78134 (15)	0.0432 (6)	
C14	0.6148 (2)	0.5387 (3)	0.70076 (17)	0.0499 (7)	
H14A	0.5514	0.5862	0.6681	0.060*	
H14B	0.5800	0.4519	0.7130	0.060*	
C15	0.7363 (2)	0.5223 (2)	0.64918 (16)	0.0440 (6)	
C16	0.7295 (2)	0.4449 (2)	0.57069 (15)	0.0421 (6)	
C17	0.6237 (3)	0.3730 (3)	0.54615 (18)	0.0631 (8)	
H17	0.5501	0.3733	0.5788	0.076*	
C18	0.6259 (3)	0.3006 (3)	0.4736 (2)	0.0720 (9)	
H18	0.5537	0.2525	0.4578	0.086*	
C19	0.7325 (3)	0.2990 (3)	0.42538 (18)	0.0636 (8)	
H19	0.7339	0.2490	0.3769	0.076*	
C20	0.8376 (3)	0.3707 (3)	0.4480 (2)	0.0706 (9)	
H20	0.9102	0.3711	0.4144	0.085*	
C21	0.8365 (3)	0.4425 (3)	0.52045 (19)	0.0633 (8)	
H21	0.9091	0.4903	0.5358	0.076*	

### Atomic displacement parameters (Å^2^)

**Table:**

	*U*^11^	*U*^22^	*U*^33^	*U*^12^	*U*^13^	*U*^23^
O1	0.0612 (12)	0.0688 (13)	0.0509 (11)	−0.0129 (10)	−0.0135 (10)	0.0076 (9)
O2	0.0428 (10)	0.0826 (15)	0.0694 (13)	−0.0102 (10)	0.0025 (9)	−0.0298 (11)
C1	0.0458 (14)	0.0501 (15)	0.0374 (13)	−0.0036 (11)	−0.0054 (10)	0.0045 (11)
C2	0.0532 (16)	0.072 (2)	0.0597 (18)	−0.0021 (14)	0.0077 (13)	−0.0045 (15)
C3	0.0565 (18)	0.108 (3)	0.067 (2)	−0.0182 (19)	0.0109 (15)	0.0035 (19)
C4	0.087 (2)	0.095 (3)	0.075 (2)	−0.043 (2)	−0.0039 (19)	0.022 (2)
C5	0.087 (2)	0.0552 (18)	0.079 (2)	−0.0176 (16)	−0.0054 (18)	0.0040 (16)
C6	0.0614 (17)	0.0513 (17)	0.0653 (18)	−0.0026 (13)	0.0027 (14)	0.0011 (14)
C7	0.0435 (13)	0.0449 (14)	0.0394 (13)	0.0032 (11)	−0.0036 (11)	−0.0012 (11)
C8	0.0337 (12)	0.0465 (14)	0.0412 (13)	0.0018 (10)	0.0023 (10)	−0.0029 (11)
C9	0.0506 (14)	0.0468 (14)	0.0431 (14)	0.0009 (11)	0.0014 (11)	−0.0050 (11)
C10	0.0537 (16)	0.0687 (19)	0.0407 (14)	0.0036 (14)	0.0055 (12)	−0.0033 (13)
C11	0.0555 (16)	0.0702 (19)	0.0466 (16)	0.0046 (14)	0.0087 (13)	0.0116 (14)
C12	0.0455 (15)	0.0500 (15)	0.0606 (17)	−0.0017 (12)	0.0069 (12)	0.0055 (13)
C13	0.0342 (12)	0.0483 (14)	0.0472 (14)	0.0014 (10)	0.0060 (10)	−0.0024 (11)
C14	0.0442 (14)	0.0488 (15)	0.0567 (16)	−0.0082 (11)	0.0024 (12)	−0.0057 (12)
C15	0.0400 (13)	0.0416 (13)	0.0503 (15)	−0.0039 (11)	−0.0025 (11)	−0.0032 (11)
C16	0.0452 (13)	0.0355 (13)	0.0457 (14)	−0.0013 (10)	−0.0016 (11)	−0.0003 (10)
C17	0.0546 (17)	0.076 (2)	0.0592 (18)	−0.0195 (15)	0.0067 (13)	−0.0189 (15)
C18	0.070 (2)	0.080 (2)	0.065 (2)	−0.0255 (17)	−0.0016 (16)	−0.0239 (17)
C19	0.087 (2)	0.0557 (17)	0.0480 (16)	−0.0083 (16)	0.0014 (15)	−0.0103 (13)
C20	0.070 (2)	0.079 (2)	0.0626 (19)	−0.0104 (17)	0.0167 (16)	−0.0213 (17)
C21	0.0504 (16)	0.0703 (19)	0.0693 (19)	−0.0135 (14)	0.0090 (14)	−0.0198 (16)

### Geometric parameters (Å, °)

**Table:**

O1—C7	1.218 (3)	C9—H9	0.9300
O2—C15	1.210 (3)	C10—C11	1.371 (4)
C1—C7	1.509 (3)	C10—H10	0.9300
C1—C6	1.520 (4)	C11—C12	1.380 (4)
C1—C2	1.528 (4)	C11—H11	0.9300
C1—H1	0.9800	C12—C13	1.388 (4)
C2—C3	1.520 (4)	C12—H12	0.9300
C2—H2A	0.9700	C13—C14	1.509 (4)
C2—H2B	0.9700	C14—C15	1.521 (4)
C3—C4	1.504 (5)	C14—H14A	0.9700
C3—H3A	0.9700	C14—H14B	0.9700
C3—H3B	0.9700	C15—C16	1.488 (3)
C4—C5	1.510 (5)	C16—C21	1.376 (4)
C4—H4A	0.9700	C16—C17	1.376 (4)
C4—H4B	0.9700	C17—C18	1.378 (4)
C5—C6	1.515 (4)	C17—H17	0.9300
C5—H5A	0.9700	C18—C19	1.355 (4)
C5—H5B	0.9700	C18—H18	0.9300
C6—H6A	0.9700	C19—C20	1.361 (4)
C6—H6B	0.9700	C19—H19	0.9300
C7—C8	1.500 (3)	C20—C21	1.374 (4)
C8—C9	1.392 (3)	C20—H20	0.9300
C8—C13	1.402 (4)	C21—H21	0.9300
C9—C10	1.378 (4)		
			
C7—C1—C6	110.5 (2)	C10—C9—C8	121.9 (2)
C7—C1—C2	111.0 (2)	C10—C9—H9	119.1
C6—C1—C2	110.0 (2)	C8—C9—H9	119.1
C7—C1—H1	108.4	C11—C10—C9	119.2 (3)
C6—C1—H1	108.4	C11—C10—H10	120.4
C2—C1—H1	108.4	C9—C10—H10	120.4
C3—C2—C1	110.9 (2)	C10—C11—C12	119.8 (3)
C3—C2—H2A	109.5	C10—C11—H11	120.1
C1—C2—H2A	109.5	C12—C11—H11	120.1
C3—C2—H2B	109.5	C11—C12—C13	122.2 (3)
C1—C2—H2B	109.5	C11—C12—H12	118.9
H2A—C2—H2B	108.1	C13—C12—H12	118.9
C4—C3—C2	111.4 (3)	C12—C13—C8	118.0 (2)
C4—C3—H3A	109.4	C12—C13—C14	118.6 (2)
C2—C3—H3A	109.4	C8—C13—C14	123.3 (2)
C4—C3—H3B	109.4	C13—C14—C15	113.7 (2)
C2—C3—H3B	109.4	C13—C14—H14A	108.8
H3A—C3—H3B	108.0	C15—C14—H14A	108.8
C3—C4—C5	111.2 (3)	C13—C14—H14B	108.8
C3—C4—H4A	109.4	C15—C14—H14B	108.8
C5—C4—H4A	109.4	H14A—C14—H14B	107.7
C3—C4—H4B	109.4	O2—C15—C16	120.3 (2)
C5—C4—H4B	109.4	O2—C15—C14	120.9 (2)
H4A—C4—H4B	108.0	C16—C15—C14	118.8 (2)
C4—C5—C6	111.1 (3)	C21—C16—C17	117.9 (2)
C4—C5—H5A	109.4	C21—C16—C15	118.0 (2)
C6—C5—H5A	109.4	C17—C16—C15	124.0 (2)
C4—C5—H5B	109.4	C18—C17—C16	120.7 (3)
C6—C5—H5B	109.4	C18—C17—H17	119.6
H5A—C5—H5B	108.0	C16—C17—H17	119.6
C5—C6—C1	111.3 (2)	C19—C18—C17	120.4 (3)
C5—C6—H6A	109.4	C19—C18—H18	119.8
C1—C6—H6A	109.4	C17—C18—H18	119.8
C5—C6—H6B	109.4	C18—C19—C20	119.8 (3)
C1—C6—H6B	109.4	C18—C19—H19	120.1
H6A—C6—H6B	108.0	C20—C19—H19	120.1
O1—C7—C8	120.3 (2)	C19—C20—C21	120.1 (3)
O1—C7—C1	120.0 (2)	C19—C20—H20	119.9
C8—C7—C1	119.7 (2)	C21—C20—H20	119.9
C9—C8—C13	119.0 (2)	C20—C21—C16	121.0 (3)
C9—C8—C7	119.2 (2)	C20—C21—H21	119.5
C13—C8—C7	121.8 (2)	C16—C21—H21	119.5
			
C7—C1—C2—C3	178.6 (2)	C11—C12—C13—C14	176.2 (2)
C6—C1—C2—C3	56.0 (3)	C9—C8—C13—C12	0.9 (3)
C1—C2—C3—C4	−56.3 (4)	C7—C8—C13—C12	−178.3 (2)
C2—C3—C4—C5	55.9 (4)	C9—C8—C13—C14	−175.6 (2)
C3—C4—C5—C6	−55.8 (4)	C7—C8—C13—C14	5.3 (3)
C4—C5—C6—C1	56.4 (4)	C12—C13—C14—C15	−116.1 (3)
C7—C1—C6—C5	−179.2 (2)	C8—C13—C14—C15	60.3 (3)
C2—C1—C6—C5	−56.3 (3)	C13—C14—C15—O2	−3.5 (4)
C6—C1—C7—O1	67.7 (3)	C13—C14—C15—C16	176.3 (2)
C2—C1—C7—O1	−54.6 (3)	O2—C15—C16—C21	−6.8 (4)
C6—C1—C7—C8	−110.8 (3)	C14—C15—C16—C21	173.3 (3)
C2—C1—C7—C8	126.9 (2)	O2—C15—C16—C17	171.7 (3)
O1—C7—C8—C9	−143.9 (2)	C14—C15—C16—C17	−8.2 (4)
C1—C7—C8—C9	34.6 (3)	C21—C16—C17—C18	0.3 (5)
O1—C7—C8—C13	35.2 (3)	C15—C16—C17—C18	−178.2 (3)
C1—C7—C8—C13	−146.2 (2)	C16—C17—C18—C19	0.1 (5)
C13—C8—C9—C10	−0.8 (4)	C17—C18—C19—C20	−0.9 (5)
C7—C8—C9—C10	178.4 (2)	C18—C19—C20—C21	1.3 (5)
C8—C9—C10—C11	0.2 (4)	C19—C20—C21—C16	−0.9 (5)
C9—C10—C11—C12	0.3 (4)	C17—C16—C21—C20	0.1 (5)
C10—C11—C12—C13	−0.2 (4)	C15—C16—C21—C20	178.6 (3)
C11—C12—C13—C8	−0.4 (4)		
